# Sleep disorders increase the risk of dementia, Alzheimer’s disease, and cognitive decline: a meta-analysis

**DOI:** 10.1007/s11357-025-01637-2

**Published:** 2025-04-11

**Authors:** Zoltan Ungvari, Mónika Fekete, Andrea Lehoczki, Gyöngyi Munkácsy, János Tibor Fekete, Virág Zábó, György Purebl, Péter Varga, Anna Ungvari, Balázs Győrffy

**Affiliations:** 1 Vascular Cognitive Impairment, Neurodegeneration and Healthy Brain Aging Program, Department of Neurosurgery, University of Oklahoma Health Sciences Center, Oklahoma City, OK USA; 2 Stephenson Cancer Center, University of Oklahoma, Oklahoma City, OK USA; 3 Oklahoma Center for Geroscience and Healthy Brain Aging, University of Oklahoma Health Sciences Center, Oklahoma City, OK USA; 4 Department of Health Promotion Sciences, College of Public Health, University of Oklahoma Health Sciences Center, Oklahoma City, OK USA; 5 International Training Program in Geroscience, Doctoral College, Health Sciences Division/Institute of Preventive Medicine and Public Health, Semmelweis University, Budapest, Hungary; 6 Institute of Preventive Medicine and Public Health, Semmelweis University, Budapest, Hungary; 7 Doctoral College, Health Sciences Division, Semmelweis University, Budapest, Hungary; 8 Department of Bioinformatics, Semmelweis University, 1094 Budapest, Hungary; 9 Cancer Biomarker Research Group, Institute of Molecular Life Sciences, HUN-REN Research Centre for Natural Sciences, 1117 Budapest, Hungary; 10 Institute of Behavioural Sciences, Semmelweis University, Budapest, Hungary; 11 Department of Biophysics, Medical School, University of Pecs, 7624 Pecs, Hungary; 12 Jozsef Fodor Center for Prevention and Healthy Aging, Semmelweis University, Budapest, Hungary

**Keywords:** Inadequate sleep, Apnoe, Sleep deficit, Cognitive decline, Aging, Circadian rhythms, Neurodegeneration, Stroke, Semmelweis Study, Sleep-disordered breathing

## Abstract

Sleep disorders, particularly insomnia and obstructive sleep apnea, are increasingly implicated as significant contributors to cognitive decline, dementia, and neurodegenerative diseases such as Alzheimer’s disease (AD) and vascular cognitive impairment and dementia (VCID). However, the extent and specificity of these associations remain uncertain. This meta-analysis evaluates the impact of common sleep disorders on the risk of developing dementia and cognitive decline. A comprehensive search of the literature was conducted to identify prospective cohort studies assessing sleep disorders and dementia risk. Studies reporting risk estimates for dementia, AD, or cognitive decline associated with obstructive sleep apnea, insomnia, and other sleep disorders (e.g., restless legs syndrome, circadian rhythm sleep disorders, excessive daytime sleepiness) were included. Meta-analyses were performed using a random-effects model to calculate pooled hazard ratios (HRs) and 95% confidence intervals (CIs). Thirty-nine cohort studies were included, with subgroup analyses showing significant associations between all-cause dementia and obstructive sleep apnea (HR 1.33, 95% CI 1.09–1.61), insomnia (HR 1.36, 95% CI 1.19–1.55), and other sleep disorders (HR 1.33, 95% CI 1.24–1.43). Obstructive sleep apnea increased the risk for AD (HR 1.45, 95% CI 1.24–1.69), though its association with vascular dementia did not reach statistical significance (HR 1.35, 95% CI 0.99–1.84). Insomnia was significantly associated with increased risk for both vascular dementia (HR 1.59, 95% CI 1.01–2.51) and AD (HR 1.49, 95% CI 1.27–1.74). This meta-analysis highlights the critical role of sleep disorders in dementia risk, emphasizing the need for early detection and management of sleep disturbances. Targeted interventions could play a pivotal role in reducing dementia risk, particularly among high-risk populations.

## Introduction

Sleep is a fundamental biological process essential for maintaining brain health, memory consolidation, and overall well-being [1, 2]. Disruptions in sleep, particularly those associated with common sleep disorders, have increasingly been recognized as key contributors to neurodegeneration and cognitive decline [3, 4]. As global populations age, dementia—including Alzheimer’s disease (AD) and vascular cognitive impairment and dementia (VCID)—has emerged as a major public health challenge, underlining the urgency of identifying modifiable risk factors to inform prevention strategies.

A growing body of evidence implicates a range of sleep disorders in the pathogenesis of dementia [5–9]. Sleep disorders are highly prevalent across populations [10] and represent a significant public health concern due to their widespread impact on physical and cognitive health. Insomnia, the most commonly reported sleep disorder, affects approximately 10–30% of the global population, with higher prevalence among older adults and women [10–13]. Sleep-disordered breathing, including obstructive sleep apnea, affects an estimated 17–22% of men and 9–17% of women in the general population, with rates increasing in individuals with obesity and other comorbidities [14–19]. Other sleep disorders include excessive daytime sleepiness, circadian rhythm sleep disorders and sleep-related movement disorders. Excessive daytime sleepiness, which is associated with poor nighttime sleep quality and is reported by up to 20% of adults [20–25]. Circadian rhythm sleep disorders, which involve misalignment between an individual’s internal circadian clock and external environmental cues, are particularly prevalent in shift workers, affecting an estimated 20–30% of this occupational group [26–28]. Among healthcare workers, the prevalence of circadian rhythm sleep disorders is especially concerning due to the demanding and irregular schedules associated with shift work, extended hours, and overnight duties. Studies indicate that up to 40% of healthcare workers experience significant circadian disruption, which not only impairs sleep quality and duration but also impacts cognitive performance, decision-making, and overall well-being. Sleep-related movement disorders, including restless legs syndrome, are reported by 5–15% of adults, with prevalence increasing with age [29, 30]. These disorders not only impact daily functioning and quality of life but are also increasingly recognized as risk factors for neurodegenerative diseases [5–9], cardiovascular conditions [31–33], and other age-related health outcomes, underscoring the need for effective management and prevention strategies.

Despite the wealth of studies investigating the strength of the relationship between sleep disorders and the risk of dementia, Alzheimer’s disease, and cognitive decline, significant inconsistencies in study design, sleep disorder classifications, and diagnostic methodologies have resulted in conflicting conclusions. Some evidence suggests that sleep disorders might serve as early biomarkers of underlying neurodegeneration, reflecting prodromal stages of Alzheimer’s disease or vascular cognitive impairment, while others highlight their potential causal role in accelerating dementia onset [34–38] through mechanisms such as inflammation [39–42], glymphatic dysfunction, and oxidative stress [43]. Besides the many links between insomnia and Alzheimer’s disease, some research suggests common genetic and psychosocial risk factors [44–46].

Building on the framework of prior meta-analyses linking sleep duration to all-cause mortality and stroke risk, this study synthesizes data from prospective cohort studies to evaluate the predictive role of specific sleep disorders—including obstructive sleep apnea, insomnia and other sleep disorders—in dementia risk. Particular emphasis is placed on Alzheimer’s disease and vascular dementia, the most prevalent subtypes of dementia. A meta-analysis offers a powerful approach to addressing these gaps by pooling data from diverse cohorts, increasing statistical power, and identifying consistent patterns across varied populations. This methodology not only clarifies the strength and direction of associations but also allows for the exploration of subgroup effects and potential modifiers. By consolidating findings across a range of sleep disorders, this study aims to resolve key uncertainties and inform public health and clinical strategies for dementia prevention.

## Methods

### Definitions

In this meta-analysis, sleep disorders examined encompassed obstructive sleep apnea, insomnia, and other sleep disorders, including excessive daytime sleepiness, sleep-related movement disorders, and circadian rhythm sleep disorders. Insomnia is defined as difficulty initiating or maintaining sleep or returning to sleep after premature awakening, while obstructive sleep apnea is characterized by interruptions in breathing during sleep. Circadian rhythm sleep disorders and other non-specific sleep issues were grouped together due to limited specific classification. In the studies analyzed, sleep disorders were identified using self-reports, clinical diagnoses, or objective measurements. Dementia diagnoses were based on recognized international criteria, such as the Diagnostic and Statistical Manual of Mental Disorders, or documented in medical records. Our analysis focused on the predictive role of these sleep disorders in the development of dementia, with Alzheimer’s disease and vascular dementia being the primary subtypes of interest.

### Search strategy and selection criteria

To identify relevant research, we performed comprehensive searches across the PubMed, Web of Science, and Cochrane Central Register of Controlled Trials (CENTRAL) databases. These databases were chosen to provide extensive coverage of biomedical and clinical literature. Our search was restricted to publications in the English language and focused on various sleep-related terms in combination with dementia and its subtypes. We also collected studies from previous meta-analyses [5–9].

The search terms included “sleep,” “sleep disorders,” “sleep quality,” “insomnia,” “obstructive sleep apnea,” (or “apnoe”, preferred in British English), “snoring,” “restless legs syndrome,” “circadian rhythm sleep disorders,” “excessive daytime sleepiness,” “dementia,” “Alzheimer’s disease,” and “vascular dementia.” These terms were systematically combined using Boolean operators to identify studies addressing the relationship between sleep disturbances and dementia. For example, we searched for combinations such as “sleep and dementia,” “insomnia and dementia,” “ssleep apnea and dementia,” “circadian rhythm sleep disorder and dementia,” “sleep disturbances and Alzheimer’s disease,” and “sleep disorders and vascular dementia”.

This iterative search strategy ensured the inclusion of studies that explored a broad spectrum of sleep-related issues and their potential associations with dementia, including both Alzheimer’s disease and vascular dementia.

**Fig. 1:**
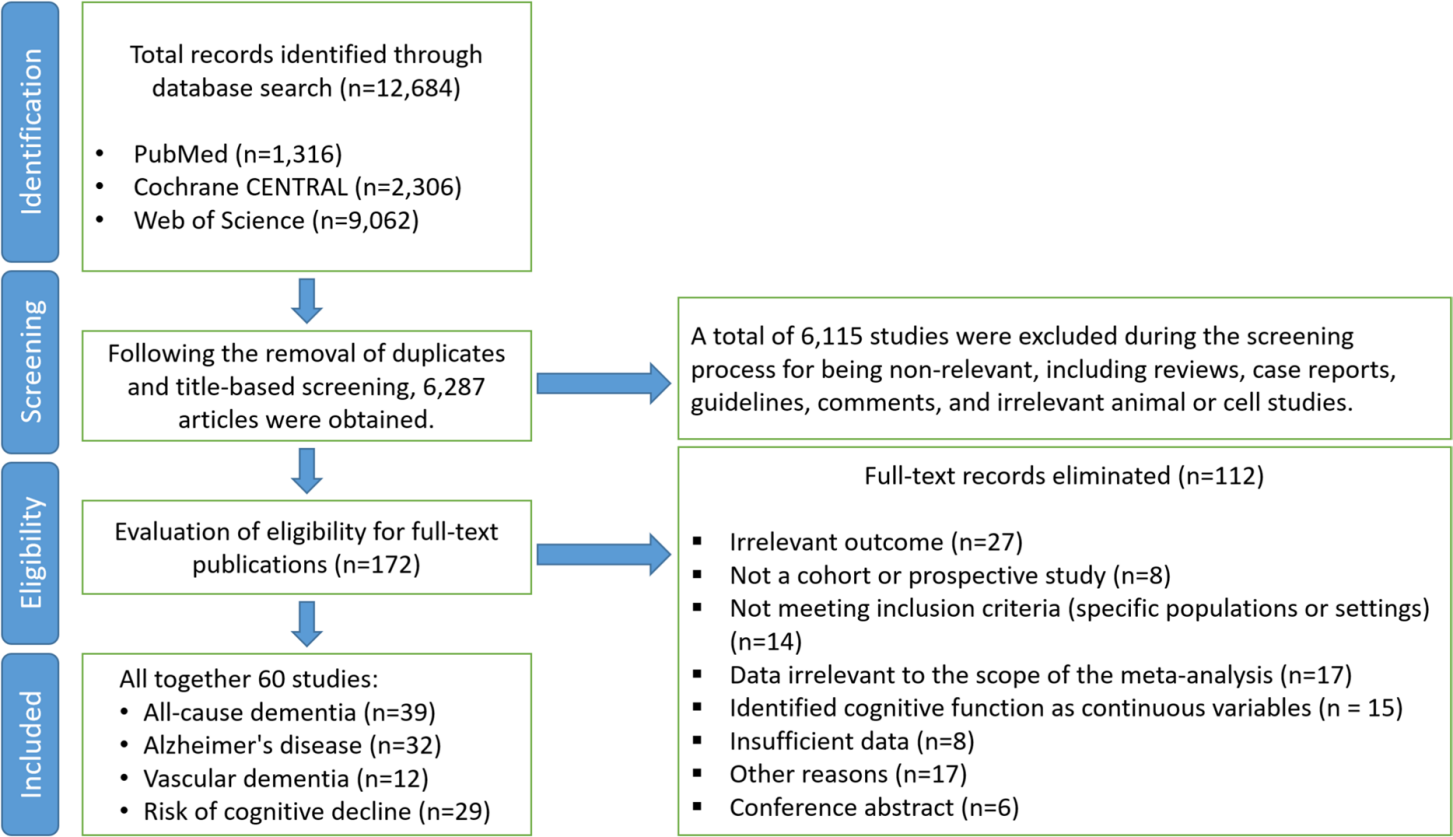
Flow diagram showing the study selection process

Two researchers independently assessed the articles for eligibility, selecting studies for inclusion in the meta-analysis based on predefined criteria. Eligible studies examined the associations between sleep disorders and dementia, with dementia diagnosed according to international diagnostic standards. Only longitudinal studies were included, provided they evaluated symptoms through self-reports, questionnaires, clinical diagnoses, or objectively measured sleep parameters, and reported effect estimates such as odds ratios (ORs), relative risks (RRs), or hazard ratios (HRs). Studies that were case reports, comments, conference abstracts, cross-sectional designs, lacked recognized diagnostic criteria for dementia, or did not provide relevant effect estimates were excluded. A detailed overview of the selection process is presented in Fig. 1.

### Data extraction

Data were independently extracted from the selected documents by two researchers (MF and AU), who then cross-verified the information. Any discrepancies were resolved through discussion until consensus was reached. The following data were collected from each study: (1) name of the first author, (2) year of publication, (3) type of research, (4) total sample size, and (5) prevalence of dementia cases during follow-up. If adjusted ORs, RRs, or HRs were reported at different levels of adjustment, the most strongly adjusted level was selected.

### Statistical analysis

The statistical analyses were conducted using the web-based platform MetaAnalysisOnline.com. To estimate pooled risk metrics, including hazard ratios (HRs) with their corresponding 95% confidence intervals (CIs), we applied a random-effects model. This approach accounts for variations across studies, enhancing the generalizability of the results. Forest plots were generated to visually represent individual study findings alongside the overall pooled estimate, facilitating interpretation of the data and identification of heterogeneity across studies.

To assess inter-study variability, we employed Cochran’s *Q* test and the *I*^2^ statistic. Cochran’s *Q* test, based on a chi-square framework, evaluated whether the observed variation in effect sizes exceeded what would be expected by chance alone. The *I*^2^ metric quantified the proportion of total variation attributable to true heterogeneity rather than random error.

### Assessment of publication bias

Potential publication bias was evaluated through visual inspection of funnel plots, which graphically display effect sizes against measures of study precision to detect asymmetrical distributions indicative of bias. For quantitative assessment, Egger’s regression analysis was performed to examine the relationship between effect sizes and their standard errors.

### Subgroup analyses

Subgroup analyses were performed separately for apnea, insomnia, and other sleep disorders. For each subgroup, we calculated pooled effect estimates and heterogeneity metrics to evaluate the specific effects on each disorder. The analyses were also performed for the combined cohort to assess aggregated effects across all sleep disorders.

## Results

### Sleep disorders and all-cause dementia

**Fig. 2:**
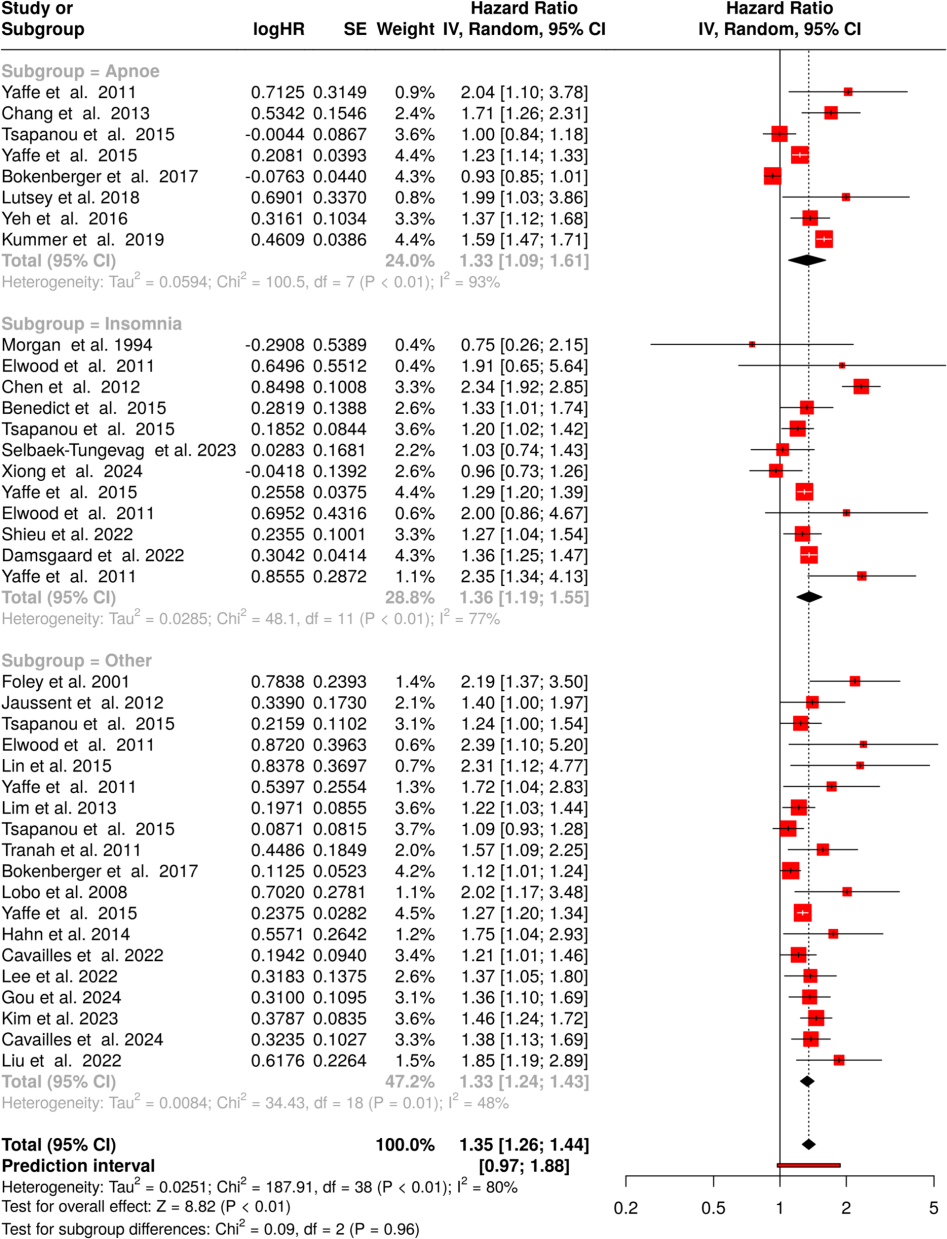
Summary of hazard ratios (HRs) for the association between apnoe, insomnia, or other sleep disorders and all-cause dementia risk across multiple studies. Each row represents an individual study, displaying the study author(s) and year of publication, the log hazard ratio (logHR), standard error (SE), weight assigned to the study in the random-effects model, and HR with 95% confidence intervals (CI). The red squares denote the HR for each study, with square size reflecting study weight. Horizontal lines indicate the 95% CI for each study’s HR. The pooled HR estimate (diamond shape) for all sleep disorders shows a significant association with an increased risk of dementia. Heterogeneity across studies is indicated by *I*^2^. Abbreviations: CI, confidence interval; HR, hazard ratio; IV, inverse variance; SE, standard error

**Fig. 3:**
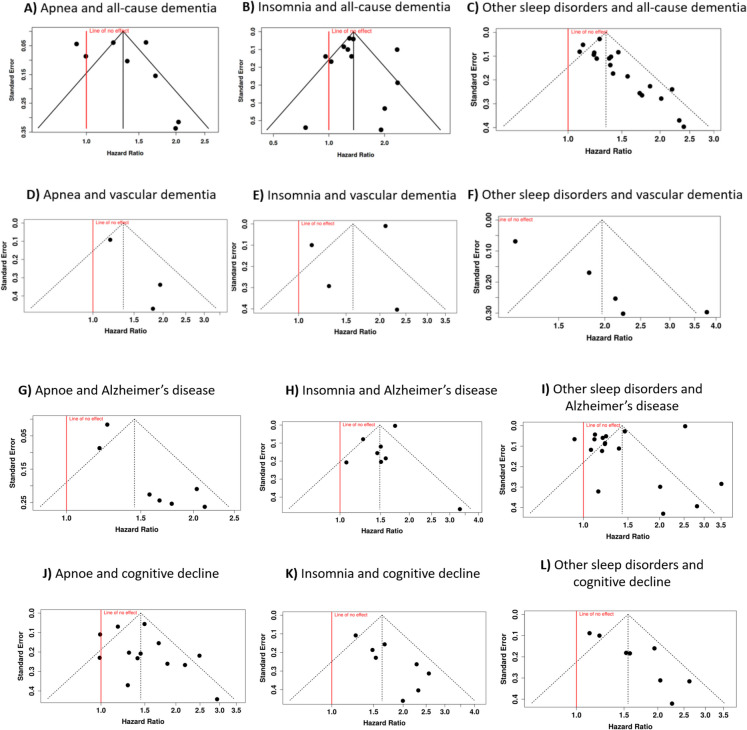
Funnel plots depicting the relationship between hazard ratios (HRs) and standard error (SE) for the association between various sleep disorders and different cognitive outcomes: all-cause dementia (**A–C**), vascular dementia (**D–F**), Alzheimer’s disease (**G–I**), and cognitive decline (**J–L**). The plots are organized into these four cognitive outcome categories, with each section examining the relationship with apnea, insomnia, and other sleep disorders. The funnel plot shape and symmetry can provide insights into potential publication bias, with asymmetrical plots suggesting the possibility of selective reporting or publication of studies

This analysis included a total of 39 cohorts, of which eight study specifically examined the association between *apnoe* and the risk of all-cause dementia (see Fig. 2, subgroup = apnoe) [47–53]. The pooled hazard ratio (HR) was 1.33 (95% confidence interval [CI] 1.09–1.61), indicating that individuals with apnoe have a statistically significant 33% higher risk of developing dementia compared to those without it. Substantial heterogeneity was observed among the studies (*p* < 0.01, *I*^2^ = 93%), showing that the effect sizes across the included cohorts were not consistent. Publication bias was evaluated using a funnel plot, which appeared symmetrical, suggesting no evidence of bias (Fig. 3A). This was corroborated by Egger’s test, which produced a non-significant intercept of 0.96 (95% CI − 4.04 to 5.96, *p* = 0.72).

Twelve cohorts were included to investigate the association between *insomnia* and the risk of all-cause dementia [47, 49, 50, 54–61]. The meta-analysis, using a random-effects model, revealed a statistically significant pooled HR of 1.36 (95% CI 1.19–1.55), indicating a 36% increased risk of dementia in those with insomnia. Notable heterogeneity was present across studies (*p* < 0.01, *I*^2^ = 77%), implying variability in effect size was influenced by differences in study design, populations, or definitions of insomnia (Fig. 2, subgroup = insomnia). The funnel plot showed no evidence of asymmetry, indicating a low likelihood of publication bias (intercept: 0.33, 95% CI − 1.58 to 2.25, *p* = 0.74, as depicted in Fig. 3B).

The third analysis in this cohort included 19 studies focusing on *other sleep disorders* [47, 49, 50, 55, 62–75]. The pooled HR was 1.33 (95% CI 1.24–1.43), demonstrating a significant association between other sleep disorders and dementia risk (Fig. 2, subgroup = other). Moderate heterogeneity was observed among the studies (*I*^2^ = 48%), suggesting that approximately half of the variability was due to differences in study methodologies or populations rather than chance. Publication bias was checked through a funnel plot, which revealed asymmetry, suggesting a potential bias (Fig. 3C). This was supported by Egger’s test, which yielded a significant intercept of 1.41 (95% CI 0.66–2.16, *t* = 3.681, *p* = 0.002), indicating a possible underrepresentation of studies with non-significant or negative results.

### Inadequate sleep and vascular dementia

**Fig. 4:**
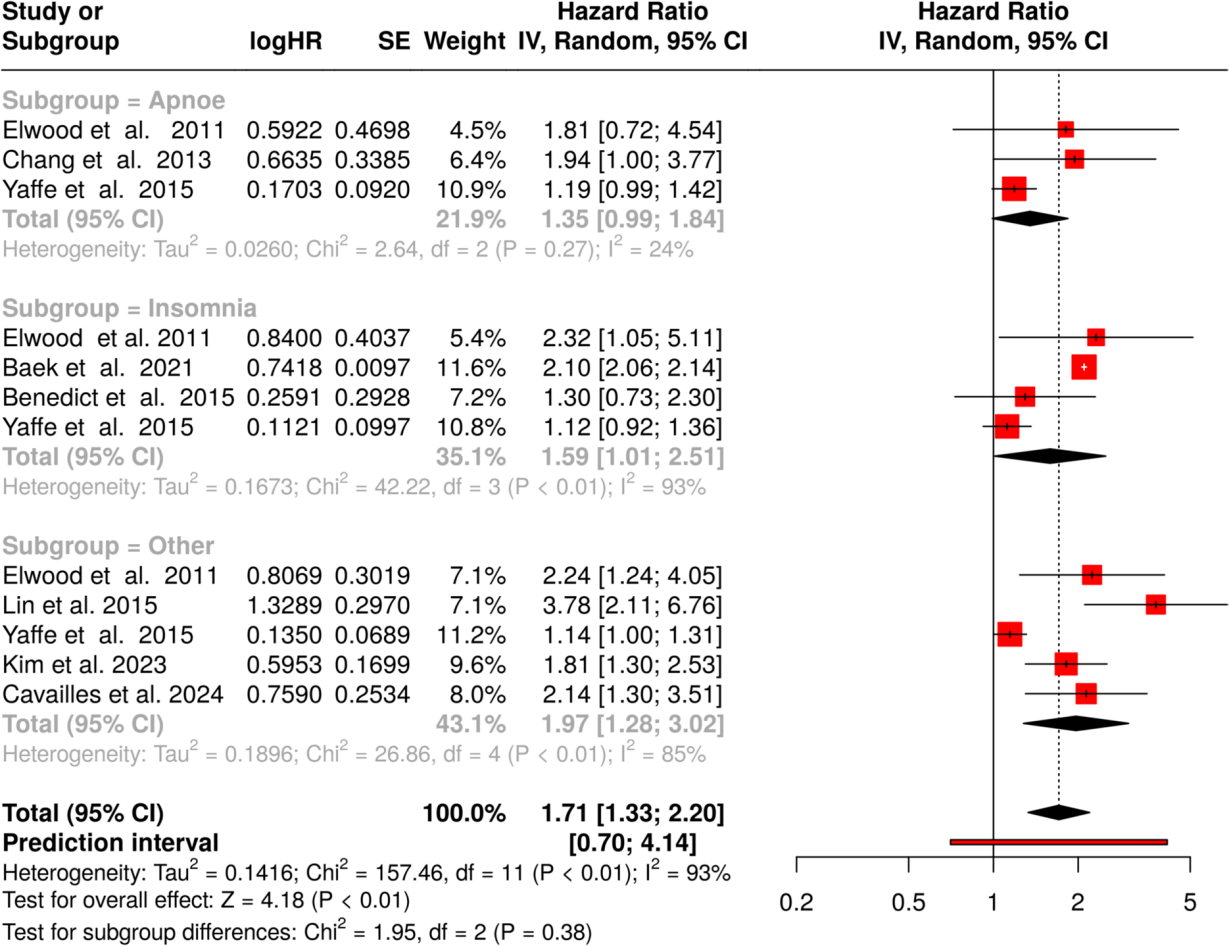
Meta-analysis exploring the relationship between sleep disorders and vascular dementia, with subgroup analyses for apnoe, insomnia, and other sleep disorders. Hazard ratios (HRs) and 95% confidence intervals (CIs) were calculated using a random-effects model. The combined analysis demonstrates a significant link between sleep disorders and vascular dementia (HR = 1.71, 95% CI 1.33–2.20). Substantial heterogeneity is reflected by an *I*^2^ value of 93%. Abbreviations: CI, confidence interval; HR, hazard ratio; IV, inverse variance; SE, standard error

Altogether 12 studies were analyzed concerning vascular dementia (Fig. 4). The meta-analysis incorporated three studies examining the relationship between *apnoe* and vascular dementia (Fig. 4, subgroup = apnoe) [48, 50, 55]. Applying a random-effects model with inverse variance methodology yielded a pooled HR of 1.35 (95% CI 0.99–1.84). These findings suggest no statistically meaningful correlation, as the confidence interval encompassed values below unity. Heterogeneity assessment revealed minimal inter-study variability, with a low *I*^2^ value (24%) confirming consistency in both magnitude and directionality of effects. Publication bias evaluation demonstrated symmetrical distribution in the funnel plot, while Egger’s test showed no significant asymmetry (intercept: 1.51, CI 0.6–2.41; *p* = 0.189) (Fig. 3D).

Four cohort studies were analyzed to determine the relationship between *insomnia* and vascular cognitive impairment. Calculations produced a pooled HR of 1.59 (95% CI 1.01–2.51), revealing a significant link between sleep disruption and elevated risk (Fig. 4, subgroup = insomnia) [50, 55, 57, 76]. The analysis yielded statistical significance, though marked heterogeneity emerged (*p* < 0.01), suggesting considerable methodological or population-based variations across studies. The *I*^2^ value of 93% indicated that inter-study differences, rather than chance, accounted for most observed variation. The funnel plot examination (Fig. 3E) showed symmetrical distribution, while Egger’s test results (intercept: − 2.6, 95% CI: − 6.76–1.56; *t* = − 1.224; *p* = 0.346) confirmed absence of publication bias.

Five investigations encompassing *other sleep disorders* were also evaluated (Fig. 4, subgroup = other) [50, 55, 64, 73, 74]. Random-effects modeling produced a pooled HR of 1.97 (95% CI 1.28–3.02), demonstrating a significant correlation. The effect test confirmed statistical significance (*p* < 0.05). Considerable heterogeneity was observed (*p* < 0.01), with an *I*^2^ value of 85%, suggesting that methodological differences primarily explained the effect estimate variability. Publication bias assessment revealed funnel plot asymmetry, confirmed by Egger’s test (intercept: 3.94, 95% CI 2.7–5.19; *t* = 6.196; *p* = 0.008), suggesting potential reporting bias among included studies (Fig. 3F).

### Insufficient sleep and Alzheimer’s disease

**Fig. 5:**
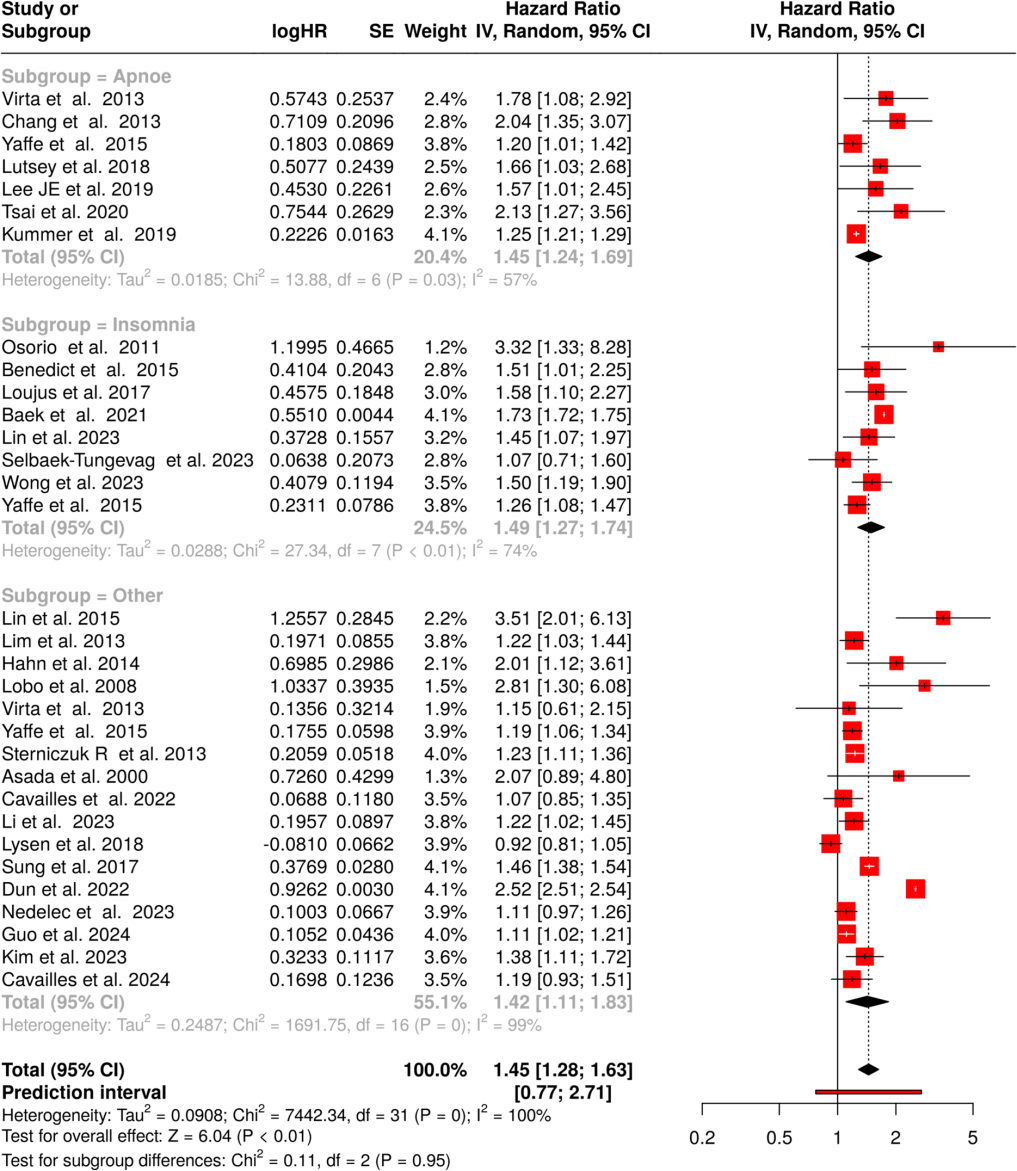
Forest plot depicting hazard ratios (HRs) for the association between sleep disorders and Alzheimer’s disease risk. The analysis includes 32 studies stratified into three subgroups: apnoe (*n* = 7), insomnia (*n* = 8), and other sleep disorders (*n* = 17). The overall pooled effect shows a significant increased risk (HR = 1.45, 95% CI 1.28–1.63). Diamond markers represent pooled estimates; squares represent individual study effects with size proportional to study weight. Abbreviations: CI, confidence interval; HR, hazard ratio; IV, inverse variance; SE, standard error

A total of 32 trials were examined related to Alzheimer’s disease (see Fig. 5). Seven studies yielded evidence for a significant correlation between *apnoe* and Alzheimer’s pathology (Fig. 5, subgroup = apnoe) [48, 50, 51, 53, 77–79]. The analysis produced a combined HR of 1.45 (95% CI 1.24–1.69; *p* < 0.05). Notable heterogeneity emerged (*p* = 0.03), with an *I*^2^ value of 57% indicating that methodological variations, rather than sampling error, accounted for the majority of inter-study differences. The funnel plot analysis revealed asymmetrical distribution (Fig. 3G), suggesting potential underrepresentation of smaller studies with non-significant results. Egger’s test confirmed this observation (intercept: 1.42, 95% CI 0.59–2.25; *t* = 3.348; *p* = 0.02).

Analysis of eight studies demonstrated a markedly elevated Alzheimer’s risk among individuals with *insomnia* (HR 1.49; 95% CI 1.27–1.74; *p* < 0.05). Substantial heterogeneity was detected (*p* < 0.01), with 74% of inter-study variability attributable to methodological differences rather than chance (Fig. 5, subgroup = insomnia) [50, 57, 58, 76, 80–82]. Publication bias assessment (Fig. 3H) revealed symmetrical distribution, supported by Egger’s test results (intercept: − 1.24, CI − 2.53–0.06; *t* = − 1.866; *p* = 0.111), suggesting negligible reporting bias.

Examination of 17 studies revealed that subjects with various sleep disturbances exhibited 42% higher Alzheimer’s risk compared to controls (Fig. 5, subgroup = other) [50, 64, 65, 68–74, 77, 83–89]. The meta-analysis generated an HR of 1.42 (95% CI 1.11–1.83; *p* < 0.05). Marked heterogeneity was observed (*p* < 0.01), with an *I*^2^ value of 99% indicating that study characteristics explained nearly all effect variations. Publication bias evaluation (Fig. 3I) demonstrated asymmetrical distribution, confirmed by Egger’s test (intercept: − 8.16, 95% CI − 11.71 to − 4.61; *t* = − 4.506; *p* < 0.01), suggesting potential underreporting of null or minimal effect findings.

### Sleep disturbance and cognitive decline

**Fig. 6:**
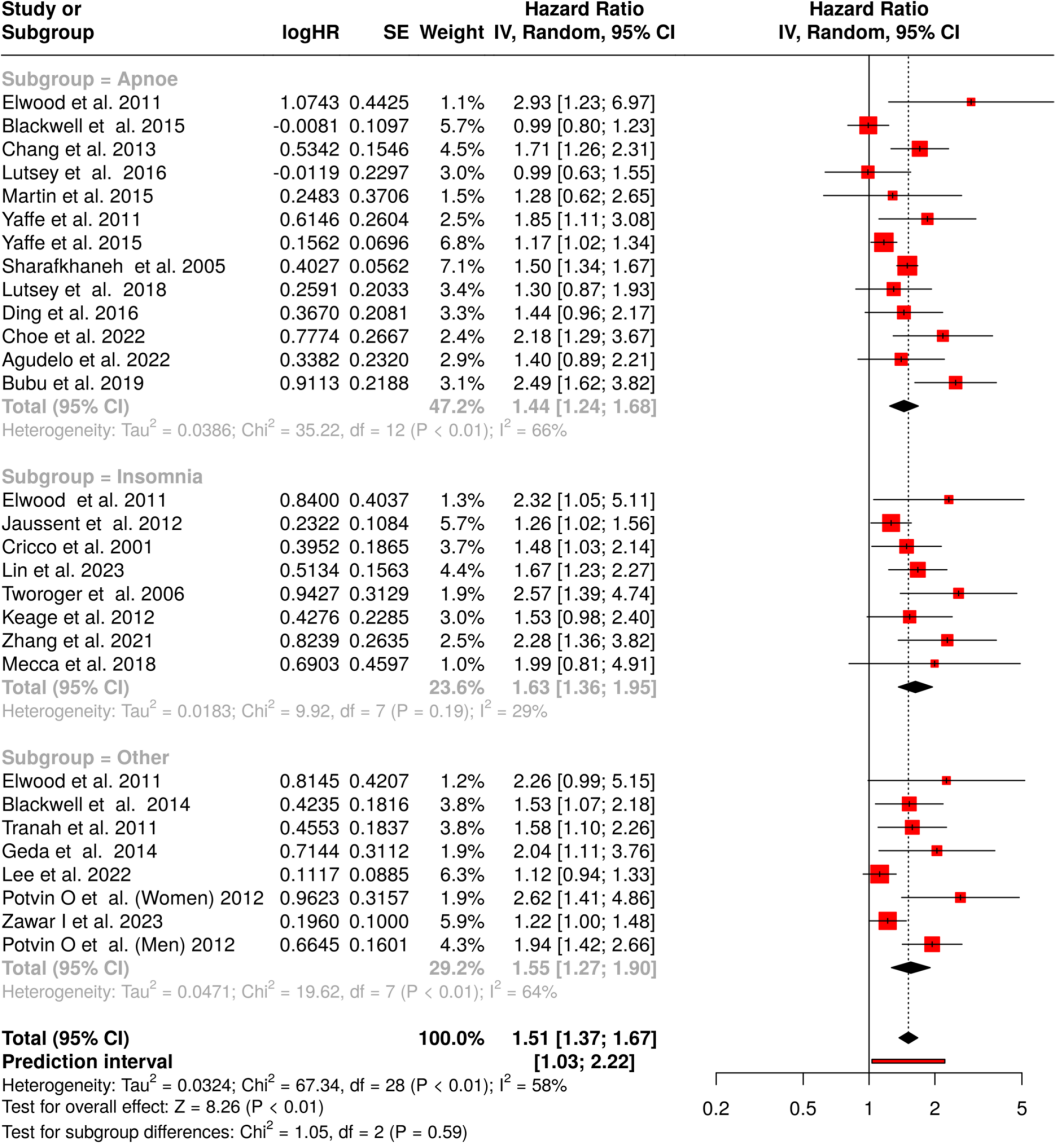
Meta-analysis results showing associations between sleep disorders and risk of cognitive decline across three categories: apnoe (*n* = 13), insomnia (*n* = 8), and other sleep disturbances (*n* = 9). The combined analysis of 30 studies demonstrates an elevated risk (HR = 1.51, 95% CI 1.37–1.67), with moderate heterogeneity across studies. Individual study estimates are shown as squares, with pooled estimates represented by diamonds. Abbreviations: CI, confidence interval; HR, hazard ratio; IV, inverse variance; SE, standard error

Altogether 29 cohorts were analyzed about cognitive functions (Fig. 6). Of these, 13 cohort studies examined the relationship between *apnoe* and cognitive decline. The meta-analysis, conducted using a random-effects model with the inverse variance method, identified a statistically significant association, with a pooled HR of 1.44 (95% confidence interval [CI] 1.24–1.68), indicating that sleep-disordered breathing is linked to an elevated risk of cognitive decline (Fig. 6, subgroup = apnoe) [47, 48, 50, 51, 55, 90–97]. Substantial heterogeneity was detected among the included studies (*p* < 0.01; *I*^2^ = 66%). A funnel plot evaluation, supported by Egger’s test (intercept: 0.87, 95% CI: − 0.74 to 2.49; *t* = 1.059; *p* = 0.312) showed no signs of asymmetry, suggesting a low likelihood of publication bias (Fig. 3J).

Eight studies were included in the meta-analysis to evaluate the link between *insomnia* and cognitive decline (Fig. 6, subgroup = insomnia) [55, 63, 98–103]. The pooled HR was 1.63 (95% CI 1.36–1.95), indicating a statistically significant increase in risk. Unlike the previous subgroup, this analysis exhibited low heterogeneity (*I*^2^ = 29%; *p* = 0.19), suggesting consistency in effect sizes across the studies. However, the funnel plot revealed asymmetry, raising concerns about potential publication bias (Fig. 3K). Egger’s test further confirmed this, demonstrating significant small-study effects (intercept: 2.18, 95% CI 1.05–3.32; *t* = 3.772; *p* = 0.009).

Finally, eight studies assessing the association between *other sleep disorders* and cognitive decline were analyzed (Fig. 6, subgroup = other) [55, 66, 71, 104–107]. The meta-analysis, using a random-effects model, identified a significant association, with a pooled HR of 1.55 (95% CI 1.27–1.90). Notable heterogeneity was observed (*p* = 0.01; *I*^2^ = 64%), indicating that the effect sizes across cohorts were not uniform in both magnitude. Funnel plot analysis suggested potential publication bias, which was further corroborated by Egger’s test (intercept: 3.01, 95% CI 1.70–4.33; *t* = 4.495; *p* = 0.004; Fig. 3L).

## Discussion

The findings of this meta-analysis provide robust evidence for an association between a range of sleep disorders—including obstructive apnea, insomnia, and other sleep disorders —and an elevated risk of dementia. These results build upon the growing recognition that sleep disturbances are not only symptoms of neurodegeneration but may also contribute causally to its onset and progression [5–9, 47–107]. This relationship underscores the critical role of sleep health in maintaining cognitive function and preventing age-related brain decline, with significant implications for both clinical practice and public health strategies.

The biological mechanisms underpinning the observed associations between sleep disorders and dementia are complex and multifactorial. Insomnia, characterized by difficulty initiating or maintaining sleep, is associated with chronic activation of the hypothalamic–pituitary–adrenal (HPA) axis [108], leading to elevated cortisol levels, systemic inflammation [39–42], and chronic oxidative stress [43], all of which exacerbate neurodegeneration [41–43, 108]. Sleep deprivation further impairs glymphatic clearance of neurotoxic proteins [109], such as amyloid-β and tau, both of which are central to Alzheimer’s disease pathology [1, 34–36, 109–111]. Obstructive sleep apnea is marked by intermittent hypoxia, hypercapnia, and sleep fragmentation. Other sleep disturbances, such as excessive daytime sleepiness and circadian rhythm sleep disorders, highlight the importance of circadian rhythm integrity for brain health. Disruption of circadian rhythms, commonly seen in shift workers, impairs restorative processes, deregulates inflammatory pathways, and disrupts the timing of critical metabolic and cognitive functions. These disturbances can lead to endothelial dysfunction [33, 37, 38, 112–114], increased blood–brain barrier permeability [115–118], impaired neurovascular coupling [119] and functional connectivity [120], and amyloid-β deposition [34, 35]—mechanisms that contribute to both Alzheimer’s disease [121] and VCID. Sleep disturbances have also been linked to systemic inflammation [39, 117, 122, 123] and metabolic dysregulation [124–126], both of which increase cerebrovascular and neuronal vulnerability. The impact of sleep disturbances in shift workers may be compounded by occupational stress, leading to chronic sleep restriction and cognitive impairment over time [127]. Collectively, these mechanistic insights demonstrate that sleep disturbances likely act through multiple, overlapping pathways to accelerate neurodegeneration and contribute to the development of dementia.

Sleep disorders may also impact the cellular mechanisms of aging [128], contributing to accelerated biological aging [129–132] and thereby increasing the risk of a range of age-related diseases, including Alzheimer’s disease and VCID. Accordingly, chronic sleep disturbances are associated with epigenetic alterations [130–133], increased oxidative stress [43, 134], systemic inflammation [39, 122], and impaired mitochondrial function [135, 136], all of which are hallmarks of aging. Supporting this concept, sleep disorders have been linked to several age-related diseases and conditions, including increased risks of stroke [137, 138], cardiovascular disease [31], and mortality [139, 140]. Furthermore, emerging evidence suggests a potential link between sleep disruption and cancer development [141–143], with mechanisms involving impaired DNA repair and enhanced tumor growth driven by chronic inflammation and hormonal imbalances. These findings underscore the broader impact of sleep disorders as accelerators of aging and highlight their role in the pathogenesis of multiple age-related diseases.

The differential effects of sleep disorders on dementia subtypes warrant further investigation. For example, the strong association of obstructive sleep apnea with vascular dementia may stem from intermittent hypoxia and its impact on cerebrovascular health, whereas insomnia’s relationship with Alzheimer’s disease is more closely tied to inflammation and impaired glymphatic clearance. Disentangling these subtype-specific effects is critical for guiding tailored prevention strategies.

The findings of this meta-analysis have significant implications for ongoing population-based research, including the Semmelweis Study, a prospective workplace cohort conducted at one of Central Europe’s largest health sciences universities [144]. This study focuses on identifying determinants of unhealthy aging, with particular emphasis on modifiable lifestyle factors [145–150], including sleep. Given its unique occupational setting [151], the Semmelweis Study provides an unparalleled opportunity to investigate the role of sleep disorders in cognitive decline and dementia risk, particularly among healthcare professionals—a group disproportionately affected by sleep disturbances due to shift work, workplace stress, and digital device overuse [152–155]. Healthcare professionals, such as doctors and nurses, are often exposed to circadian misalignment caused by rotating shifts and irregular work schedules [152–155]. These disruptions are well-documented contributors to insomnia, excessive daytime sleepiness, and poor sleep quality, which, as shown in this meta-analysis, significantly elevate dementia risk. By incorporating comprehensive assessments of sleep duration, quality, and disorders, the Semmelweis Study is uniquely positioned to provide longitudinal insights into the cumulative effects of sleep disturbances on cognitive health in a high-risk population.

Emerging evidence suggests that sleep disturbances may also serve as early biomarkers of neurodegeneration [156]. Conditions such as insomnia and disrupted circadian rhythms are often observed in the prodromal stages of Alzheimer’s disease, potentially reflecting underlying amyloid-β or tau pathology [157]. Identifying whether specific sleep disorders consistently precede cognitive decline could establish them as preclinical indicators of dementia, enabling earlier diagnosis and intervention [157]. The Semmelweis Study is well-positioned to explore these dynamics longitudinally, shedding light on the temporal relationships between sleep disturbances and neurodegeneration.

Sleep disorders frequently coexist with other chronic conditions, such as cardiovascular disease, diabetes, and depression, which independently increase dementia risk. Understanding how these comorbidities interact with sleep disturbances to accelerate cognitive decline is a critical area for future research. Furthermore, integrating genetic and epigenetic analyses—such as the role of circadian regulation genes (e.g., CLOCK and BMAL1) or APOE variations—could elucidate individual variability in sleep-dementia pathways and support the development of personalized interventions.

Moreover, the Semmelweis Study can examine how occupational stress and lifestyle factors, such as physical inactivity or prolonged screen exposure, interact with sleep disturbances to amplify dementia risk. For instance, by evaluating circadian rhythm disruption in shift workers, the study can elucidate the extent to which misalignment between biological rhythms and occupational demands contribute to cognitive decline over time. These findings could guide the design of workplace interventions to improve sleep health and mitigate long-term dementia risk in vulnerable occupational cohorts.

The Semmelweis-EUniWell Workplace Health Promotion Program, which targets the same population, is well-positioned to translate these findings into actionable interventions. Programs that focus on improving sleep hygiene—such as reducing exposure to blue light from screens, implementing stress management strategies, and promoting regular sleep schedules—could be tailored to address the specific needs of shift workers and high-stress professionals. By incorporating workplace-based interventions, such as rotating shift schedules aligned with natural circadian rhythms and protected rest periods, the Semmelweis-EUniWell Workplace Health Promotion Program could provide effective tools to reduce the adverse effects of occupational sleep disturbances on brain health.

This meta-analysis has several strengths, including its comprehensive synthesis of data from diverse cohorts, robust methodology, and focus on a range of sleep disorders. By differentiating between various sleep disturbances, this study provides a nuanced understanding of the unique contributions of insomnia, obstructive sleep apnea, and other disorders to dementia risk. However, several limitations must be acknowledged. A notable limitation of this meta-analysis is the variability in study populations, diagnostic criteria, and sleep disorder assessment methods. Many of the included studies relied on self-reported sleep measures, which are subject to recall bias and misclassification. Participants may underreport or overestimate their sleep problems due to individual differences in perception or recall limitations. Self-reported measures may also fail to capture the full extent of sleep disturbances. Objective assessments, such as actigraphy or polysomnography, would provide more accurate evaluations of sleep patterns and disturbances. While self-report remains a widely used method in epidemiological studies due to its practicality, future investigations should prioritize objective sleep assessments to strengthen the reliability of findings and minimize potential bias. Additionally, significant heterogeneity was observed across studies, reflecting variations in study design, sleep disorder definitions, and follow-up duration.

Our analysis of publication bias using funnel plots revealed asymmetry, particularly in studies examining the relationship between other sleep disorders and dementia risk. This suggests a potential underrepresentation of studies with non-significant findings, which may skew the pooled effect estimates. Egger’s test confirmed a small-study effect in certain subgroup analyses, indicating that studies with smaller sample sizes and non-significant results might be less likely to be published. While we attempted to mitigate this by including all available cohort studies, future research should focus on addressing publication bias through pre-registered studies and the inclusion of null findings to provide a more balanced perspective on the association between sleep disturbances and dementia.

Building on the insights from this meta-analysis, future research should explore the causal pathways linking specific sleep disturbances to dementia using advanced imaging and biomarker-based approaches. Longitudinal studies are needed to assess the cumulative impact of sleep disorders on cognitive decline while accounting for occupational stress and lifestyle factors.

The question of whether treating sleep disorders can mitigate dementia risk remains an important avenue for future research. Evidence suggests that continuous positive airway pressure (CPAP) therapy for obstructive sleep apnea can improve cognitive performance, particularly in executive function and memory [158–164]. However, long-term data on whether CPAP prevents or delays dementia onset remain limited. Similarly, cognitive behavioral therapy for insomnia (CBT-I) has been shown to enhance sleep quality and reduce systemic inflammation, both of which may help preserve cognitive function. Future studies should examine the extent to which early interventions, such as CPAP, oral appliances, or pharmacological approaches, reduce dementia risk in high-risk populations. Given the increasing recognition of sleep disturbances as modifiable risk factors, integrating sleep health into dementia prevention strategies could have significant public health implications.

In conclusion, this meta-analysis highlights a significant association between sleep disorders and dementia risk, emphasizing the critical role of sleep health in preventing neurodegeneration. The findings underscore the importance of early identification and management of sleep disturbances, particularly in high-risk populations such as healthcare professionals. The Semmelweis Study, with its focus on occupational cohorts and sleep health, is uniquely positioned to address these knowledge gaps, offering valuable insights into the interplay between sleep disorders, occupational stress, and cognitive decline. By informing targeted interventions [165] and public health strategies, this work provides a foundation for reducing dementia risk and promoting healthy brain aging in vulnerable populations.
